# Cycles of circadian illuminance are sufficient to entrain and maintain circadian locomotor rhythms in *Drosophila*

**DOI:** 10.1038/srep37784

**Published:** 2016-11-24

**Authors:** Eunjoo Cho, Ji Hye Oh, Euna Lee, Young Rag Do, Eun Young Kim

**Affiliations:** 1 Department of Brain Science, Ajou University School of Medicine, 164 Worldcup-ro, Suwon, 16499, Republic of Korea; 2 Chronic Inflammatory Disease Research Center, Ajou University School of Medicine, 164 Worldcup-ro, Suwon, 16499, Republic of Korea; 3 Department of Chemistry, Kookmin University, Seoul, 136-702, Republic of Korea; 4 Neuroscience Graduate Program, BK21 Plus Program, Department of Biomedical Sciences, Ajou University School of Medicine, 164 Worldcup-ro, Suwon, 16499, Republic of Korea

## Abstract

Light at night disrupts the circadian clock and causes serious health problems in the modern world. Here, we show that newly developed four-package light-emitting diodes (LEDs) can provide harmless lighting at night. To quantify the effects of light on the circadian clock, we employed the concept of circadian illuminance (CIL). CIL represents the amount of light weighted toward the wavelengths to which the circadian clock is most sensitive, whereas visual illuminance (VIL) represents the total amount of visible light. Exposure to 12 h:12 h cycles of white LED light with high and low CIL values but a constant VIL value (conditions hereafter referred to as CH/CL) can entrain behavioral and molecular circadian rhythms in flies. Moreover, flies re-entrain to phase shift in the CH/CL cycle. Core-clock proteins are required for the rhythmic behaviors seen with this LED lighting scheme. Taken together, this study provides a guide for designing healthful white LED lights for use at night, and proposes the use of the CIL value for estimating the harmful effects of any light source on organismal health.

All living creatures on earth have an endogenous clock to anticipate environmental changes such as light/dark cycles. The circadian clock system has evolved to have a period of about 24 h, over which changes in behavior and physiology occur^1^,^2^. This clock needs to be reset and synchronized every day for accuracy and coordination. A resetting cue is referred to as a *zeitgeber*, which means “time-giver” in German, and sunlight is the most prominent one.

The molecular mechanism underlying the circadian clock is evolutionarily conserved from unicellular organisms to humans^3^,^4^. Studies using *Drosophila* as a model system have unveiled core-clock genes and their functions in the molecular clockwork^5^. The circadian clock operates at the cellular level and is mainly driven by transcriptional and translational feedback loops. In the core loop of *Drosophila*, transcription factors dCLOCK (dCLK) and CYCLE (CYC) form a heterodimer to activate the expression of core-clock genes, such as *period* (*per*) and *timeless* (*tim*), in addition to other clock and clock-controlled downstream genes. Newly synthesized proteins PER and TIM undergo timely posttranslational modifications leading to daily oscillation of levels, activity, and subcellular localization^5^,^6^. Translocation of PER/TIM into the nucleus turns off circadian transcription by inhibiting the activity of dCLK/CYC, and the eventual degradation of PER/TIM enables another round of transcription the next day^5^,^6^. This underlying molecular clockwork drives changes in circadian behavior, such as locomotor activity levels of *Drosophila*, over the course of each 24-h period.

Until artificial lights were developed, natural sunlight was the only available light source. Although artificial lighting systems have allowed the humans to have an active night life, light at night (LAN) has become a serious health problem because it provides inaccurate information about the time of the day to our internal clock. Indeed, the circadian clock is often disrupted in humans, and numerous reports have indicated an association between circadian disruption and disease, including sleep, mental, and metabolic disorders^7^,^8^,^9^. The causative role of circadian disruption in human health problems has been confirmed by numerous studies of shift workers. Shift workers have a higher risk of developing diseases such as diabetes, cardiovascular disease, and obesity^8^. The risk of breast cancer is also higher in shift workers and, indeed, LAN increased the growth of human breast cancer xenografts in a rodent model^10^,^11^. The immune system and internal circadian system interact so closely that susceptibility to infection also oscillates daily, and immunity is often impaired in individuals with abnormal circadian rhythmicity^12^. Although we acknowledge the serious risk of LAN and circadian disruption to human health, it is very difficult to change the modern lifestyle. Furthermore, some facilities require lights for all 24 h of the day. For example, emergency rooms and intensive care units (ICUs) need constant lighting to monitor the health of patients. Because severe disruption of the circadian clock and sleep loss affect many processes, such as the immune response, hormone secretion, and mental ability, exposure to constant light ultimately impairs the recovery of patients in the ICU^13^,^14^. Limiting the use of LAN, therefore, is not the solution for these kinds of problems. Rather, we must make efforts to develop lighting systems that are healthful for organismal circadian physiology.

Toward this end, we need to understand which characteristic of light is the most important in controlling the circadian clock. The circadian clock is most sensitive to the wavelengths that correspond to blue light. In mammals, melatonin secretion is a well-studied circadian output that is suppressed by light^15^,^16^. Suppression of melatonin secretion was examined using lights of different wavelengths, and suppression was found to be greatest at a wavelength of ~450–470 nm^17^. Although this wavelength is included in the visual range of the spectrum, visual perception through classic retinal photoreceptors (rods and cones) is dispensable for circadian light responses^18^,^19^. Instead, the photosensitive retinal ganglion cells (RGCs) innervating the suprachiasmatic nucleus (SCN) are responsible for these responses^20^. Maximal sensitivity of RGCs and opsin-based photopigments occurs in the blue region of the light spectrum^20^. These notions led to the discovery of melanopsin, which is expressed in subsets of RGCs, as a novel photoreceptor for the circadian light response^21^,^22^. Light signals perceived by the visual system are transduced to the SCN in the hypothalamus and entrain the circadian oscillators. Master-clock neurons in the SCN orchestrate the timing of most circadian behavior and physiology. In *Drosophila, cryptochrome* (*cry*) encodes a blue-light receptor for circadian entrainment^23^,^24^. CRY is expressed not only in compound eyes, the organs dedicated to visual perception, but also in most clock neurons in the brain^25^. CRY undergoes conformational changes upon light exposure, allowing it to bind to TIM^26^,^27^. This leads to the ubiquitination-dependent degradation of TIM and CRY via F-box protein JETLAG and the proteasomal pathway^28^,^29^,^30^. Light-induced degradation of CRY and TIM is a key regulatory mechanism in the circadian oscillation of clock proteins. Under conditions of constant light (LL), flies display arrhythmic behavior, likely due to constant degradation of CRY and TIM.

The need for stronger and energy efficient light has led to the invention of light-emitting diodes (LEDs), which have been widely used because they are long-lived, eco-friendly, inexpensive, and efficient. We have previously developed easily tunable four-package LED lights that made it possible to control the correlated color temperature (CCT) within the white range as a function of time^31^. Tunable four-package LED lamp easily produces light with various spectral power distributions (SPDs). Taking advantage of this tunable LED lamp, we aim to establish a healthful lighting system that will not disturb the circadian clock. Development of a healthful lighting system requires a method to quantitatively characterize and validate the health effects of the lighting system. However, no figure of merit has been determined to quantify the effects on the circadian rhythm. In our previous study, we introduced circadian illuminance (CIL) as such a figure of merit to indicate the extent to which light affects the circadian clock^31^. Visual illuminance (VIL) measures the amount of visible light that illuminates a surface and can be expressed in lux (lx). Similar to VIL, CIL can be defined as the portion of light that is capable of influencing the circadian clock system, and can be expressed in units of biolux (blx). CIL is calculated from VIL, weighted using wavelength functions determined by melatonin secretion^31^,^32^. CIL values represent brightness in the circadian sense, independent of the specific type of light source used. Decreasing the CIL of light without compromising VIL is desirable for nighttime lighting, but a light with this property has never been developed.

Thus, we sought to examine the validity of CIL as a measure of circadian influence *in vivo* and to use this metric to develop a healthful lighting scheme for practical use. Here, using tunable four-package LED lights, we have shown that lights with high CIL value have greater potential to disrupt the circadian clock, and 12-hour cycles of high and low CIL-value light (conditions hereafter referred to as CH/CL) can generate daily locomotor rhythms like those produced by typical 12-hour light/dark (LD) cycles. Moreover, this lighting scheme can generate molecular rhythms of core-clock proteins in flies. We have also shown that daily locomotor rhythms generated by CH/CL cycles are dependent on functional core-clock machinery and can be re-entrained to a 6-h phase shift in the CH/CL light cycle. Taking these data together, we propose that CH/CL light cycles produced using tunable four-package LED lights constitutes a healthful lighting system for use at night, and moreover, we suggest using the CIL value as a standard to represent the influence of light on organismal circadian health.

## Results

### Constant LED light with high CIL disrupts circadian rhythms faster than low CIL light

**Figure 1:**
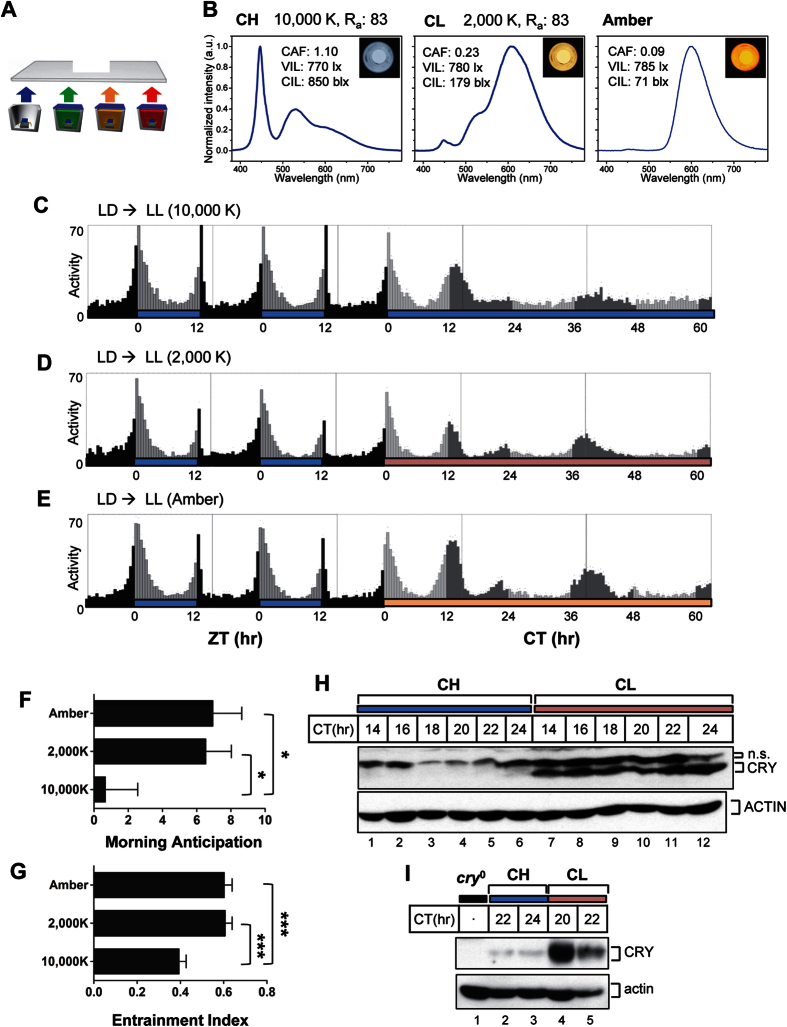
White LEDs with high circadian illuminance (CIL) abolish circadian rhythm more rapidly than LEDs with low CIL. (**A**) Schematic illustration of color-tunable and high-color quality four-package white LEDs using B semiconductor-type LEDs and long-wavelength pass dichroic filter (LPDF)-capped monochromatic green, amber, and red phosphor-converted LEDs. (**B**) Spectral power distribution of CH (cool white, 10,000 K), CL (warm white, 2,000 K), and amber LEDs are shown. (**C** to **E**) Locomotor activity of *w*^1118^ flies under different light conditions. *w*^*1118*^ flies were entrained by daily cycles of 12 h 10,000 K light followed by 12 h complete darkness (LD). After 4 days of entrainment, flies were exposed to constant light (LL) from the three kinds of LED. Light blue, pink, and amber horizontal bars indicate 10,000 K, 2,000 K, and amber LED lights whose qualities are shown in (**B**). Black horizontal bars indicate complete darkness. Each vertical bar represents average relative activity during a 30-min bin. During the LD cycles, light gray vertical bars represent activity during lights-on periods and black vertical bars represent activity during lights-off periods, whereas during LL conditions, light gray vertical bars represent locomotor activity during subjective day and dark gray vertical bars represent activity during subjective night. ZT, zeitgeber time; ZT0, lights-on time; CT, circadian time; CT0, subjective lights-on time. (**F**) Morning anticipation values and (**G**) entrainment indices were calculated for the three different LED light conditions. The numbers of flies used for the analyses shown in (**C–G**) are 31, 30, and 31 for 10,000 K, 2,000 K, and amber, respectively. Error bars denote standard error of the mean (SEM). Data were compared by pairwise Student’s *t*-test. *p < 0.05, ***p < 0.0001. (**H**,**I**) Flies were exposed to constant CH light or CL light, and heads were collected at indicated time points. Protein extracts were analyzed by immunoblotting with anti-CRY (Rb2) antibody. Note that CRY levels were higher in CL light than in CH light at every time point tested. n.s. denotes for non specific band.

The concept of CIL was introduced to represent the strength of the circadian effect of different lighting conditions^31^. Additional details regarding the measurement of vision and the circadian performance of light are described in Supplementary Information. We hypothesized that CIL, rather than VIL, is the critical factor in perturbation of a circadian clock system *in vivo*. To test our hypothesis, we applied light from two different white LEDs that differed in CIL but were similar in VIL, using *Drosophila melanogaster* as an *in vivo* model system. Light performance was finely adjusted using previously developed tunable four-package white LEDs (Fig. 1A)^31^. CCT is commonly used to describe the perceived color of white artificial light sources. Cool white (CCT 10,000 K) and warm white (CCT 2,000 K) LED lights showed similar VIL values of approximately 770 lx, yet drastically different CIL values of 850 blx and 179 blx, respectively (Fig. 1B). The color-rendering index (CRI, R_a_) must be used to indicate how well the true color of objects is described by white artificial light sources. Here, 10,000 K and 2,000 K white lights showed the same R_a_ value of 83. This means that our four-package white LEDs provide higher color quality than typical commercial lamps, such as a cool white fluorescent lamp (CCT of 6,500 K, R_a_ of 77) or a warm white high-pressure sodium lamp (CCT of 2,000 K, R_a_ of 20). Amber LED light having a similar VIL value was also applied because it has the lowest CIL value (71 blx) that this LED lamp can produce, although it is not white light. SPDs of the 10,000 K, 2,000 K, and amber LEDs showed clear differences in the intensity of blue light in each (Fig. 1B). Flies were entrained to LD cycles of 12 h of 10,000 K white LED and 12 h of complete darkness for 4 days to generate synchronized rhythmicity. They were then maintained in LL conditions using 10,000 K, 2,000 K, or amber LED lights, and locomotor rhythms were analyzed (Fig. 1C–E). In LD cycles, flies exhibited clear bimodal peaks of locomotor activity centered around the lights-on/-off transitions, designated the morning and evening peaks, respectively. When flies were transferred to the three conditions of constant light, locomotor activity was significantly suppressed, and both morning and evening peaks disappeared, showing arrhythmic patterns consistent with previous reports^33^,^34^,^35^. Among the three conditions of constant LED light, 10,000 K white LEDs, having the highest CIL, had the strongest effect in eliminating morning and evening activity peaks (Fig. 1C). In 2,000 K white and amber LED light, morning and evening locomotor activity peaks persisted until day 2, but the subjective evening peak was slightly delayed (Fig. 1D and E). We have quantified these effects by calculating the entrainment index (EI) and morning anticipation (MA) on day 2 of LL. The EI measures the proportion of peak activity to total activity of the day^36^. The circadian oscillator induces an anticipatory activity increase right before the onset of morning or evening. MA measures the ratio of activity before the onset of morning to activity of midnight^37^. Thus, EI and MA can serve as measures of the strength of circadian oscillations. Both the EI and MA values were significantly lower in the 10,000 K LED condition than in either the 2,000 K or amber LED conditions (Fig. 1F and G). The EI and MA values manifested under conditions of 2,000 K white LED light were similar to those in amber LED conditions. These data indicate that 10,000 K white LED light has a much stronger effect than 2,000 K or amber light in disrupting the circadian oscillations, which supports our notion that CIL is critical when considering the harmful effect of LEDs on the circadian clock system. One interesting observation is that amber light has an effect similar to that of 2,000 K light, even though amber light has a lower CIL value (71 blx vs. 179 blx for 2,000 K light).

In *Drosophila*, CRY is a photoreceptor for the circadian clock system and shows maximal absorption of light at blue wavelengths. Light-dependent degradation of CRY plays a crucial role in entraining the circadian oscillator, and constant degradation of CRY is responsible for the arrhythmic locomotor behavior observed in constant light conditions^38^. Thus, we have examined the effects of constant exposure to 10,000 K and 2,000 K white LED light on CRY levels by western blot analysis. CRY was not detected throughout the subjective night period in the constant 10,000 K exposure condition (Fig. 1H, lanes 1 to 6 and Fig. 1I, lane 2 and 3). In sharp contrast, strong CRY bands were observed under conditions of constant 2,000 K exposure, likely resulting from reduced CRY degradation (Fig. 1H, lanes 7 to 12 and Fig. 1I, lane 4 and 5). The severity of perturbation of locomotor rhythms by two different white LEDs correlated with the extent of CRY degradation and the level of CRY protein. Based on these observations, we used 2,000 K and 10,000 K white LED lights for further experiments.

### Alternation of white LED light with high and low CIL values generates rhythmic locomotor behavior and molecular oscillation of core-clock proteins

To maintain a strong circadian rhythm, it is best to cycle between strong natural light and complete darkness. However, the modern lifestyle does not allow an absolute LD cycle. Because we have found that 10,000 K and 2,000 K white LED light with different CIL values have different effects on CRY degradation, we sought to examine whether cycles of LED light with high and low CIL values generate circadian rhythms in flies. If so, 2,000 K white LED would serve as a healthy LAN source, preserving the organism’s circadian rhythm. We applied high CIL 10,000 K light as the subjective daytime signal and low CIL 2,000 K light as the subjective nighttime signal at alternating 12 h intervals, denoted as CIL high/CIL low (CH/CL). Using this scheme, we attempted to mimic an LD cycle in the circadian sense.

**Figure 2:**
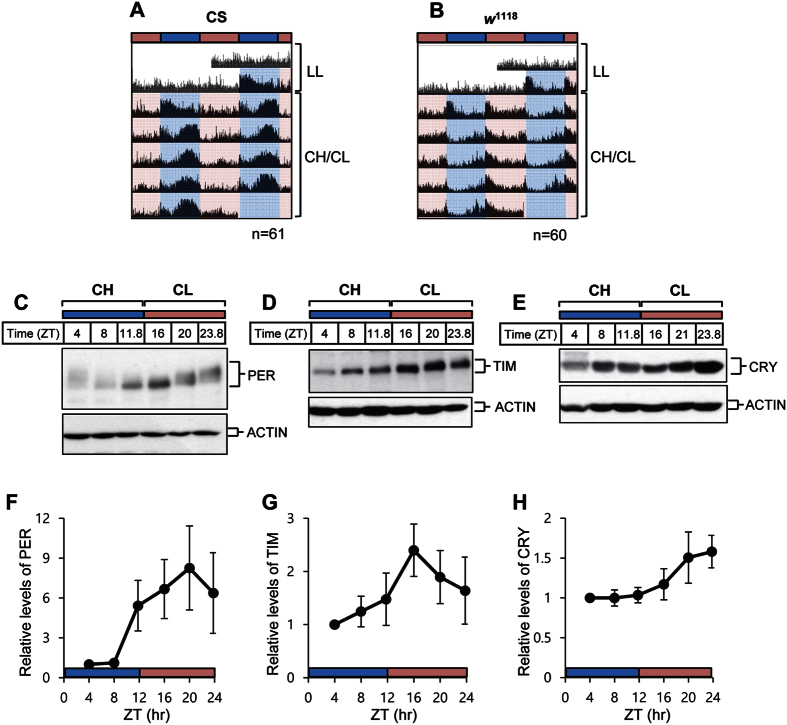
12 h:12 h cycles of white LED light with high and low circadian illuminance (CIL) produce robust behavioral and molecular rhythms. Flies were exposed to conventional constant light (LL) conditions for 2 days and then were maintained on 12 h:12 h cycles of 10,000 K white LED light with high CIL (CH) and 2,000 K white LED light with low CIL (CL) for 11 days. (**A** and **B**) Averaged actograms of flies of the indicated genotypes are shown. Each row of the actogram was double-plotted. The numbers of flies used for the analyses were 61 (CS), 60 (*w*^1118^). Light blue and pink horizontal bars indicate CH and CL, respectively. (**C** to **H**) Head extracts from CS flies on day 5 of the CH/CL cycle were obtained and processed for immunoblotting with anti-PER (**C**), anti-TIM (**D**), and anti-CRY (**E**) antibodies. Actin served as a loading control. Relative levels of PER (**F**), TIM (**G**), and CRY (**H**) were determined by measuring band densities using Image J software. Three replicates were used for the calculation. Values represent mean ± SEM.

**Figure 3:**
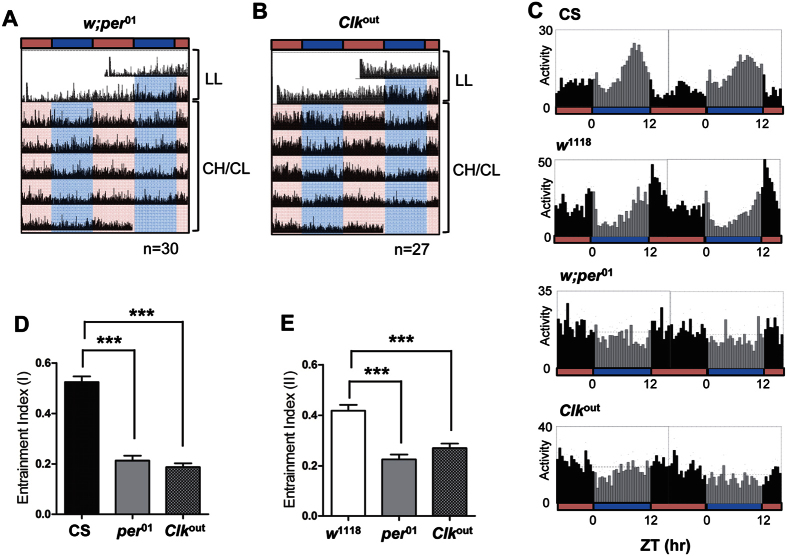
CH/CL-cycle–induced circadian locomotor activity is dependent on core circadian-clock genes. Flies were exposed to conventional constant light (LL) conditions for 2 days and then maintained on 12 h:12 h cycles of 10,000 K white LED light with high CIL (CH) and 2,000 K white LED light with low CIL (CL) for 11 days. (**A** and **B**) Averaged actograms of clock mutants *w; pe*r^01^ (**A**) and *Clk*^*out*^(**B**) flies are shown. Each row of the actogram was double-plotted. The numbers of flies used for the analyses were 30 (*pe*r^01^) and 27 (*Clk*^*out*^). Light blue and pink horizontal bars indicate CH and CL, respectively. (**C**) Daily average activity profiles of flies of indicated genotype on days 5 and 6 of 12 h:12 h CH/CL cycles are shown. The numbers of flies used for the analyses were 61 (CS), 60 (*w*^1118^), 30 (*pe*r^01^) and 27 (*Clk*^*out*^). Light gray vertical bars represent activity during the CH period and black vertical bars represent activity during the CL period. (**D** and **E**) Entrainment indices were calculated for flies of each genotype under CH/CL cycles using time windows ZT6–12 (**D**) and ZT9–15 (**E**). ZT, zeitgeber time. Values represent mean ± SEM and are compared by pairwise *t*-test. ***p < 0.0001.

Flies were first exposed to typical LL conditions under >2000-lx white fluorescent light to eliminate any existing circadian rhythm, and subsequently to CH/CL cycle conditions. We have tested two different control strains of flies, Canton S (CS) and *w*^1118^. Whereas both strains showed arrhythmic locomotor activity under typical LL conditions, locomotor activity peaks appeared on day 2 of CH/CL cycle, and the rhythms of locomotor activity persisted until the end of CH/CL cycles (Fig. 2A and B). Nonetheless, the daily locomotor activity distribution of CS and *w*^1118^ flies in CH/CL cycles differed from those in typical LD cycles. In CH/CL cycles, CS flies manifested a unimodal peak of activity that occurred around ZT10 (ZT0 means when CH light is on) preceded by a gradual increase in anticipatory activity. Because the locomotor activity peak appeared in the afternoon during the CH/CL cycle we refer to this as the afternoon peak. In contrast, *w*^1118^ flies exhibited a unimodal peak of activity that occurred primarily at the transition from CH to CL light, with a gradual increase in anticipatory activity before the transition (Fig. 2A and B, Fig. 3C). These data indicate that some characteristics of the light might affect behavioral outcomes in these flies, and that strain-specific variation may exist.

Daily oscillation in the levels and phosphorylation states of core-clock proteins are the hallmark of molecular clockworks^39^,^40^,^41^,^42^,^43^. Because strong locomotor rhythmicity was maintained under CH/CL conditions, we examined whether timely molecular oscillation of core-clock proteins also occur under this condition. CS flies were entrained to CH/CL cycles, and head extracts were prepared every 4 h on day 5 for western analysis. PER proteins manifested daily fluctuations in level and phosphorylation status, as indicated by changes in electrophoretic mobility (Fig. 2C). Hypo-phosphorylated isoforms showed greater mobility in the gel and appeared in subjective early night (at ZT11.8 and ZT16), whereas hyper-phosphorylated isoforms showed decreased mobility and appeared in the subjective late night/early day (at ZT23.8 and ZT4) (Fig. 2C). Quantification of PER band intensity clearly showed daily oscillation of PER levels (Fig. 2F). TIM also exhibited oscillations in protein abundance (Fig. 2D and G). These PER and TIM molecular rhythms are comparable to those seen under conventional LD conditions, as described in previous reports^42^,^44^,^45^. Because the VIL value of light used in the CH/CL condition was similar to that of light used under conventional LL conditions, we concluded that the strong LD–condition-like molecular oscillation of PER and TIM seen under CH/CL conditions is generated by differences in the CIL of the light. Because light of different CIL affects CRY degradation differently (Fig. 1H and I), we examined CRY levels in flies exposed to CH/CL cycles. CRY showed strong oscillations, with low levels under CH conditions and high levels under CL conditions (Fig. 2E and H). Given that CRY is sensitive to blue light, the difference in CIL values in CH/CL cycle conditions resulted in the difference in CRY levels. Blue-light-mediated degradation of CRY together with TIM leads to degradation of PER, because TIM normally binds to and stabilizes PER^26^,^27^,^45^,^46^. Therefore, we think that cycling levels of CRY during CH/CL cycles may drive the oscillation of TIM and PER levels, resulting in quasi-normal locomotor rhythms.

Intriguingly, although the behavioral patterns of *w*^1118^ and CS flies are very different, the molecular oscillation pattern of *w*^1118^ is very similar to that of CS, indicating that core-clock oscillation is similarly regulated in two fly strains by CH/CL cycles (Fig. S1).

### Core circadian-clock machinery is required for the rhythmicity induced by CH/CL cycles

It is possible that the rhythmic behavior observed under CH/CL conditions is a direct behavioral response to the change in LED lighting. To distinguish whether the rhythmic behavior was generated by the circadian clock machinery or was solely a light-induced response, we assayed locomotor behaviors of core-clock mutant flies in CH/CL light conditions. Null mutations of *per* (*pe*r^01^) and *dClk* (*Clk*^out^) completely disrupt oscillation of core-clock proteins and produce arhythmicity in flies^47^,^48^,^49^,^50^. If the rhythmic locomotor behavior observed under CH/CL conditions depends solely on a response to light that does not require circadian-oscillator function, these clock mutants should manifest the same behavior as control flies. However, these circadian clock mutants did not show any rhythmic behavior under CH/CL conditions, whereas both *w*^1118^ and CS flies displayed rhythmicity with a 24-h period (compare Fig. 2A and B with Fig. 3A and B).

To quantify the rhythmicity of locomotor behavior, we determined the EIs for CS and *w*^1118^ flies and compared them with those of *pe*r^01^ and *Clk*^*out*^flies. Because CS and *w*^1118^ flies manifest peak activity at slightly different times of the day, we chose two windows of time. As expected, CS and *w*^1118^ flies showed significantly higher EI values than those of circadian-clock mutant flies (Fig. 3C–E). These results indicate that CH/CL cycles induce circadian rhythmicity through the functions of the core circadian machinery.

### Cyclic change in CIL, not the absolute CIL level, is important to drive circadian rhythm

**Figure 4:**
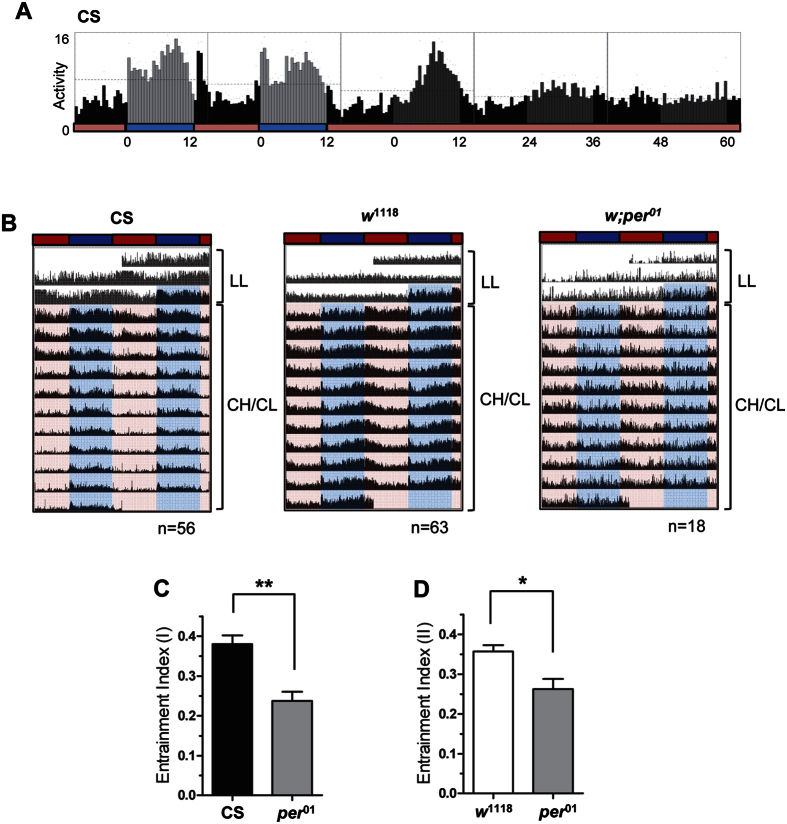
Difference in CIL, not absolute CIL level, is important to drive circadian rhythmicity in CH/CL cycles. (**A**) Flies were first exposed to constant light (LL) for 2 days and then entrained to CH/CL cycles for 7 days, followed by constant CL conditions for 7 days. Daily activity profiles of CS flies from day 6 of CH/CL to day 3 of CL/CL are shown. Activity peaks became lower and disappeared beginning on day 3 of constant CL. The numbers of CS flies used for the analyses were 56. (**B**) Averaged actograms of flies of indicated genotypes are shown. Each row of the actogram was double-plotted. The numbers of flies used for the analyses were 56 (CS), 63 (*w*^1118^), and 18 (*pe*r^01^). VIL and CIL of 10,000 K (CH) light are increased to 1,570 lx and 1,734 blx, respectively. Those of 2,000 K (CL) light are increased to 1,470 lx and 338 blx, respectively. Dark blue and red horizontal bars indicate brighter CH and CL, respectively. Flies were first exposed to constant light for 2 days and then exposed to 12 h:12 h cycles of brighter CH/CL. Whereas CS and *w*^*1118*^ flies show circadian rhythmicity, *pe*r^01^ flies show no rhythmic pattern, which is similar to observations made previously for CH/CL cycles with low light intensity. (**C** and **D**) Entrainment indices were calculated for flies of each genotype under brighter CH/CL cycles using time windows ZT6–12 (**C**) and ZT9–15 (**D**). ZT, zeitgeber time. Values represent mean ± SEM and are compared by pairwise *t*-test. **p < 0.001. *p < 0.05.

Our finding that CH/CL cycles can generate circadian rhythmicity is surprising because the photoreceptor encoded by *cry* has exquisite sensitivity to light^51^. Flies can be entrained to light levels as low as 0.03 nW/cm^2^, which is much lower than the brightness of either CH or CL light, 0.113 mW/cm^2^ and 0.114 mW/cm^2^, respectively. However, this sensitivity value is in terms of overall light intensity (similar to VIL) and not the limit of sensitivity of CRY to blue-light intensity, at least in terms of CIL. Thus, we wondered if the CIL value of CL light is below the limit of blue-light sensitivity of CRY, and therefore, may mimic true darkness in the circadian sense in our CH/CL cycle conditions. To test this, CS flies were first entrained to CH/CL conditions for 7 days and then exposed to constant CL conditions for 7 days (Fig. 4A). If CL light was perceived as complete darkness in the circadian sense, persistent rhythmicity would be expected under constant CL condition following CH/CL cycling, as it is under DD conditions following LD cycles. However, CS flies showed dampened locomotor activity on day 2 of constant CL and completely arrhythmic locomotor activity beginning on day 3 of constant CL condition. This dampening was also observed in the molecular rhythm of PER obtained from head extracts on day 2 of constant CL condition (Fig. S2). Taking these data together, we conclude that the CL condition *per se* does not function as darkness even in the circadian sense. If that is the case, then how do CH/CL cycle produces robust locomotor activity rhythm anticipating times of the day? Our data raise the possibility that the difference, not the absolute value, in blue-light intensity might be critical for circadian entrainment. Because CH has a stronger effect on CRY degradation than CL (Fig. 1H), the oscillation in CRY level resulting from CH/CL condition might be sufficient to transmit entraining cues to the clock machinery and drive robust rhythmicity in flies. To further support this idea, we doubled the VIL and CIL values for CH and CL lights. Again, VIL values for CH and CL remained similar, 1570 lx and 1470 lx, respectively. The CIL values were increased from 850 blx to 1734 blx for CH (10,000 K) and from 179 blx to 338 blx for CL (2,000 K). Although the absolute level of CIL increased throughout the CH/CL cycle, flies still show a circadian behavioral rhythm in this new CH/CL cycle (Fig. 4B). CS and *w*^1118^ flies showed higher EI values than those of *pe*r^01^ flies (Fig. 4C and D). It is surprising that exposure to bright light, such as new CL (1470 lx, 338 blx) during subjective night does not disturb the circadian behavior if flies are exposed to light that is brighter (1570 lx, 1734 blx) in terms of CIL value, during the subjective day. From our observations, we suggest that the difference in CIL, not the absolute level, is important to drive circadian rhythm. This observation raises the possibility that CL light can be used as a harmless light source in combination with CH light during the day.

### CH/CL cycle provides a strong entrainment signal for circadian rhythmicity

A key feature of the circadian clock is that it can be entrained by environmental cues and synchronize internal time to the environment. If flies are exposed to a sudden shift in LD cycle, they can re-synchronize circadian behaviors to the LD cycle regimen. The speed of re-entrainment depends on the intensity of daylight^52^.

**Figure 5:**
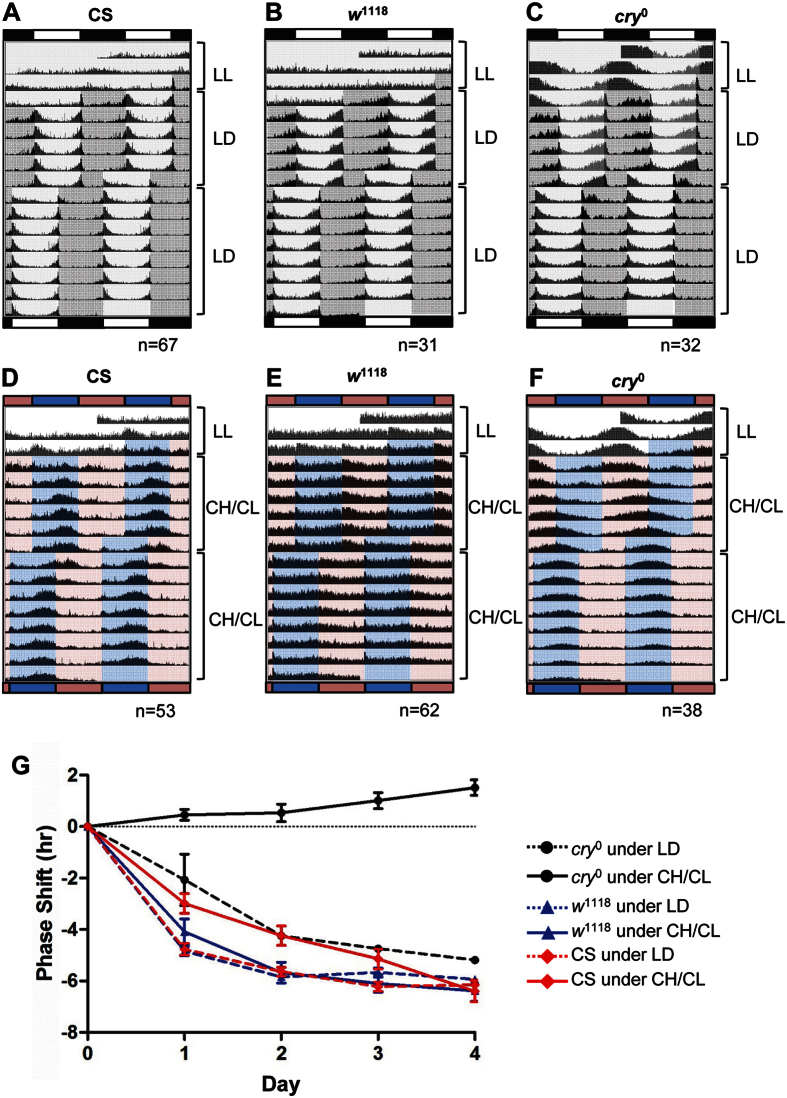
Wild-type flies can be re-entrained to a 6-h phase shift in the CH/CL cycle. (**A** to **F**) Averaged actograms of flies of indicated genotypes are shown. Each row of the actogram was double-plotted. Flies were first exposed to constant light (LL) for 3 days and then entrained to either a conventional laboratory LD cycle (**A–C**) or CH/CL cycle (**D–F**) for 6 days. The properties of CH and CL lights used for this experiment were the same as those of the 10,000 K and 2,000 K lights in Fig. 1B. On day 7, the lights-on time was advanced by 6 h for both LD and CH/CL. The number of flies used for the LD shift was 67 (CS), 31 (*w*^1118^), and 32 (*cry*^*0*^). For the CH/CL shift, 53 (CS), 62 (*w*^1118^), and 38 (*cry*^*0*^) flies were used. (**G**) Day 0 indicates the day before the shift and day 1 indicates the first day of the shift. The time of peak activity of each fly for days 0, 1, 2, 3, and 4 was determined using ActogramJ after smoothing. The difference in peak time between day 0 and the indicated day was calculated, averaged, and plotted for each condition. In the LD phase shift, CS flies completely adjusted to the new LD regimen by day 3 of the shift, whereas it took more than 4 days for *cry*^*0*^ flies to advance the phase by 6 h. In the CH/CL phase shift, CS flies shifted phase gradually and reached 6 h of phase shift on day 4. *cry*^*0*^ flies did not follow the phase shift of CH/CL cycles.

We wondered whether the CH/CL cycle could act as a strong entraining signal like the LD cycle. To test the ability of the CH/CL cycle to re-entrain flies, we exposed flies to CH/CL cycles for 7 days, and then advanced the CH/CL cycle by 6 h. Flies were also subjected to a 6-h shift in LD cycle for comparison. After a 6-h advance of the light-cycle regimen, CS flies showed a 6-h advance in phase of activity rhythms both in LD and CH/CL cycles (Fig. 5A and D). To quantitate the speed of phase resetting, we calculated hours of phase shift for four days following the light shift, as previously described^53^. Because CS flies only show a unimodal afternoon peak during CH/CL cycles, we determined the magnitude of phase shift of the afternoon peak and compared it with that of the evening peak during LD cycles. In LD cycles, the phase of the evening peak in CS flies was completely reset to the new light scheme on day 3 of the shift (Fig. 5A and G). The speed of resetting was slightly slower in CH/CL cycles, where it takes 4 days for CS flies completely reset to new light scheme (Fig. 5G). *w*^*1118*^ flies show faster resetting to phase shift than CS flies in CH/CL cycles (Fig. 5E and G). As previously reported for *cry*^*b*^ mutants^54^, *cry*^*0*^ flies re-entrained to a 6-h LD shift, albeit more slowly than control CS flies (Fig. 5C). Intriguingly, *cry*^*0*^ flies do not respond at all to change in CH/CL light scheme and behave like completely blind flies in CH/CL cycles (Fig. 5F and G). The activity peaks of *cry*^*0*^ flies seem to free-run with a period of 24.9 h, which is close to the previously reported free-running period of the *cry* mutant (Fig. 5F)^38^,^55^. This result indicates that the phase shift of locomotor activity of CS flies requires the function of CRY in CH/CL cycles, and both CRY-dependent and -independent light-input pathways may play roles in circadian entrainment in a conventional white-light cycle regimen^54^. Taken together, these data show that CH/CL cycles are perceived as constant light to CRY-independent input pathways and, moreover, the CH/CL cycle generates circadian rhythms solely dependent on CRY function. No contribution from CRY-independent input pathways in CH/CL conditions may explain the slightly slower of phase resetting of CH/CL shift compared to LD shift (Fig. 5G).

## Discussion

Light at night in the modern world is sometimes called “light pollution” and threatens human health. Although it is widely acknowledged that blue-wavelength light is the culprit behind the harmful effects of LAN, there is no good measure of the circadian effects of a particular quality of light. To choose proper lighting, we should be able to determine this information for any lighting system. We have introduced CIL as a figure of merit representing the ability of light to influence the circadian clock. By simply alternating CIL while maintaining constant VIL, we have successfully established a system of continuous lighting capable of entraining the clock. Thus, this study has described the promising possibility that use of white LED light at night may be less harmful to circadian physiology if the CIL is properly adjusted.

Based on our results, CIL may serve as a valuable standard to express the quality of light in terms of its circadian effect^31^. Lighting, therefore, could be carefully selected based on this value and the needs of the user. For example, use of lights with high CIL during the day and low CIL during the night might be an ideal lighting system for human health. For convenience in practical use, an adjustable lighting system might be ideal. In this study, we used a white LED lamp combined with a semiconductor-type blue LED and green, amber, and red phosphor-converted LEDs (pc-LEDs), which can be easily tuned from cool white (bluish white) to warm white (reddish white) by controlling the current applied to each primary monochromatic blue, green, amber, and red LED^31^. Also, this light can be programmed to change gradually, similar to natural sunlight, and create an LD cycle in the circadian sense.

Light can influence animal behavior in two ways. One is by regulating the endogenous circadian clock, which then regulates behaviors according to the time of day. The other is through a path independent of the circadian clock system. The latter is called a masking effect of the light because it may conceal the circadian behavior^56^. We argue that our CH/CL condition regulates animal behavior through core circadian oscillators rather than a masking effect for the following reasons. First, mutant flies that lack functional circadian oscillators did not show any rhythmic locomotor behavior, suggesting a requirement for core-clock proteins in this CH/CL-cycle-induced rhythmicity (Fig. 3A). Second, the core-clock proteins PER and TIM displayed strong molecular-oscillation patterns during CH/CL cycles (Fig. 2C and D; Fig. 2F and G). Third, phase shifts in locomotor activity show transient states that are due to the adjustment of the circadian clock, whereas changes induced by masking are expected to be more immediate (Fig. 5). Taken together, these data show that CH/CL cycles produce rhythmic behavior through circadian-oscillator function.

Although the CH/CL cycle condition renders flies strongly rhythmic both behaviorally and molecularly, the daily locomotor activity pattern of CS flies under these conditions was quite different from those under a standard 12 h:12 h LD cycle (Fig. 1C and Fig. 2A). In conventional laboratory LD cycles, flies show a bimodal morning and evening activity peak occurring at lights-on/off transitions. It has been well established that morning and evening oscillator neurons are responsible for these activities^57^,^58^,^59^. Whereas CS flies exhibit a typical bimodal morning and evening locomotor activity peak in LD cycles, these flies showed a unimodal activity peak in the afternoon and no obvious morning and evening peaks under CH/CL cycle conditions. Although LD cycles have long been used to entrain the endogenous clock in laboratory settings, the abrupt transition between light and dark is not like natural day-to-night transition^60^. Moreover, natural daylight differs from constant laboratory light in that many characteristics of the daylight change over time. It has been reported that a novel afternoon (A) peak in addition to the morning and evening peaks appear when flies are kept under conditions of natural light^60^. Intriguingly, the pattern of an A peak is very similar to the afternoon peak of CS flies in our CH/CL cycle condition. Absence of an A peak in laboratory LD cycles is attributed to the masking effect of strong light that suppresses the locomotor activity^56^,^60^,^61^. Consistently, when flies are subjected to skeleton photoperiods, which provide only 10-min pulses of light at each of the two transition points, the A peak reappears in the afternoon^62^. This is further supported by a study showing that daytime activity is increased under low levels of illumination^63^. Thus, we think that the appearance of the afternoon peak in CS flies under conditions of CH/CL cycle may be caused by the decreased masking effect of the weak intensity of CH/CL lights (770 lx/780 lx) vs. commonly used laboratory lights (~2000 lx). Disappearance of morning and evening peaks under CH/CL conditions may be due either to malfunction of morning and evening oscillators or to the problems in output pathways to behavioral activity. Further studies are required to test these possibilities.

Under CH/CL cycle condition, two control strains, CS and *w*^1118^ flies, exhibited a strong unimodal locomotor activity peak, yet the phases were different (Fig. 2A and B; Fig. 3C). It was previously reported that laboratory control flies express one of two *tim* alleles, which confer different light sensitivity on TIM. The *ls-tim* allele encodes a less light-sensitive TIM^64^, whereas the *s-tim* allele encodes a natural light-sensitive TIM protein. We have sequenced our control flies and confirmed that CS flies contain the *ls-tim* allele and *w*^1118^ flies have the *s-tim* allele. The combination of decreased illuminance and decreased light sensitivity in CS flies may collectively explain the absence of a masking effect in CS but not in *w*^1118^ flies where locomotor activity is suppressed in the afternoon, delaying activity onset (Fig. 2A). We also think that relatively weak rhythms of locomotor behavior of *w*^1118^ flies (Fig. 3D, compare EIs of CS and *w*^1118^ flies) is likely due to increased masking effects because the pattern of molecular oscillations of PER in *w*^1118^ is almost identical to that of CS (Fig. S2) and *w*^*1118*^ flies are re-entrained quickly to the 6-h phase advance (Fig. 5E and G).

Complete darkness is easily achieved in the laboratory for circadian entrainment. However, it never becomes completely dark in nature because of moonlight and starlight, even if there is no artificial light. Because moonlight dose not suppress melatonin secretion or induce phase shift, it is considered darkness in the context of the circadian system^65^,^66^. However, it has also been reported that dim light, such as moonlight, can affect some aspects of circadian rhythms both in flies and mammals, indicating that dim light is not equivalent to darkness even in the circadian context^67^,^68^. Then, what is the low limit of light intensity that the circadian system can sense? Researchers have long been curious about this and have tried to determine what constitutes effective darkness for the circadian clock in several model systems^52^,^69^,^70^,^71^. In flies, circadian rhythm was shown to be extremely sensitive^70^,^71^. Dim light equivalent to starlight appears to be the low limit for preservation of eclosion rhythms^71^. Nonetheless, most studies examined light sensitivity of the circadian system in terms of the total amount of light, which corresponds to VIL. However, our study clearly shows that the response of the circadian clock may change depending on SPD, even for lights of similar intensity. The lower limit of light intensity for circadian perception, if such a limit exists, should be determined by CIL, which indicates the true efficacy of circadian effects. Our study indicates that CL light is not equivalent to effective darkness because prolonged exposure to CL light disrupts both behavioral and molecular rhythms. Even amber light, which has the lowest possible CIL value that our lamp can produce, appears to disrupt circadian rhythmicity with constant exposure. Thus, unfortunately, we cannot determine the lowest limit of light in terms of CIL at this stage, and future study, together with a more refined lamp that can produce extremely dim blue light, is required.

Although we have not achieved true darkness in the circadian sense with CL light, CH/CL cycles are very effective in generating circadian locomotor rhythmicity. Because the VIL of CH/CL cycle is constant, this light regimen might be considered equivalent to conventional laboratory LL. However, our results clearly indicate that CH/CL cycles are different from conventional LL in that CH/CL generates circadian rhythmicity, which depends on core circadian genes. Moreover, even brighter CH and CL lights can produce circadian rhythmicity if differences in CIL exist between subjective day and night (Fig. 4B–D). Our data strongly support the possibility that the absence of relative difference in blue light intensity, rather than the absolute level is detrimental to disruption of circadian rhythm.

In an effort to improve lighting conditions for human circadian physiology, some studies use light filters to completely block wavelengths shorter than about 500 nm^72^. This filtered light is effective in preserving the normal cycling of melatonin secretion. However, filtered light is not ideal at night because it is not white light and exhibits poor color quality. CRI measures the ability of light to reveal the colors of objects. In our study, the CRI value of CH is higher than that of a fluorescent lamp, and the CRI of CL is higher than that of high-pressure sodium lamp. This information indicates the high color quality of both CH and CL conditions used here. In addition, the LED light we used is a white light, which includes all of the wavelengths of visual light and does not impair work performance at night. Taken together, we think that our lighting system may serve as a good alternative to systems now in use, especially for hospital ICUs. Because this form of light does not disrupt the circadian clock, which affects many aspects of health, including immunity, patients would be expected to recover more quickly from serious illnesses. In addition to the beneficial effects on patients, use of this lighting system would provide ICU doctors and nurses with an improved visual environment to provide better nighttime medical care to patients without loss of visual acuity and color discrimination.

## Materials and Methods

### Lighting

The four-package white LEDs with 10,000 K (CH) and 2,000 K (CL) were operated by controlling the applied current of each primary blue LED and green, amber, and red pc-LEDs while maintaining the same total applied current. The color coordinates of 10,000 K (CH) and 2,000 K (CL) white SPDs were (0.275, 0.264) and (0.525, 0.420), respectively, in the 1931 CIE color space. The CRI (R_a_) indicates how well a lighting system describes the true color of objects and is also optimized by tuning the relative intensity of each primary blue LED and green, amber, and red pc-LED while maintaining a similar CCT value. The R_a_ of 10,000 K (CH) and 2,000 K (CL) white SPDs is the same. The CRI of the 10,000 K (CH) white SPD is greater than that of the fluorescent lamp SPD (6,500 K, CRI of 77) and the CRI of the 2,000 K (CL) white SPD is much greater than that of a high-pressure sodium lamp SPD (2,000 K, CRI of 20). This means that the four-package white LEDs provide the capability to tune CCT and CIL combined with high color quality^31^,^32^. Detailed information on the fabrication of tunable white LEDs is provided in SI 2. For conventional lighting, a white fluorescent lamp with >2,000 lux was used.

### Fly stocks and behavioral assays

Canton S (CS), *w*^1118^, *pe*r^01^, *Clk*^out^, and *cry*^*0*^ strains of *D. melanogaster* were used. The locomotor activity of flies was measured as previously described using the *Drosophila* Activity Monitoring system from Trikinetics^73^. Young male adult flies were used for the analysis and subjected to the different LED light conditions described above. The incubator temperature was kept constant at 25 °C for all experiments. The daily activity patterns were analyzed using FaasX software, which was generously provided by F. Rouyer (CNRS, France). ActogramJ software was used for the phase analysis and actogram analysis^74^.

### Data analysis

Morning anticipation (MA) was calculated as previously described^37^. Briefly, the average activity between ZT17 and ZT19.5 was subtracted from average activity between ZT21.5 and ZT24. To quantify entrainment, we calculated the entrainment index (EI), which is the ratio of the activity during peak times (6 h) to the overall 24 h, as previously described^36^. Because the peak time differs depending on genotype and light scheme, we have chosen different windows of peak activity for each calculation. For LL conditions after LD entrainment, the time window from ZT12 of to ZT18 of day 2 of LL is selected because an evening peak normally occurs within that window of time in LD cycles. Peak time windows selected for CH/CL cycles is from ZT6 to ZT12 for CS and from ZT9 to ZT15 for *w*^1118^. Because *pe*r^01^ and *Clk*^out^ flies do not show obvious activity peaks, activity in two time windows (ZT6–12, ZT9–15) were calculated for the comparison.

For the calculation of phase shift, the activity pattern of each fly is smoothed by convolution with Gaussian kernel. Then, the peak time point was determined manually and used to calculate phase shift between days. The values calculated for individual flies were averaged for analysis and plotting^53^,^74^.

### Immunoblotting

Protein extracts were prepared from flies as previously described^75^. Flies were collected by freezing at the indicated times, and total fly head extracts were prepared using 0.1 M HEMG buffer: 10 mM HEPES, pH 7.5, 50 mM KCl, 10% glycerol, 5 mM Tris-HCl. PER and TIM were resolved on 5% gels and CRY on 8% gels. Approximately 20 to 40 ug of total protein was loaded on gels. Anti-dCRY antibodies were generated against bacterially purified pGEX4T-CRY, which contains the full-length CRY coding sequence (AbFrontier Inc, Korea). The primary antibodies used are anti-CRY (1:3000, Rb2), anti-TIM (1:2000, Rb1), and anti-PER (1:3000, Rb1)^75^. Quantification of band intensity was performed using ImageJ software (NIH).

## Additional Information

**How to cite this article**: Cho, E. *et al*. Cycles of circadian illuminance are sufficient to entrain and maintain circadian locomotor rhythms in *Drosophila. Sci. Rep.* **6**, 37784; doi: 10.1038/srep37784 (2016).

**Publisher's note:** Springer Nature remains neutral with regard to jurisdictional claims in published maps and institutional affiliations.

## Supplementary Material

Supplementary Information
