# Supplementary material for: Cycles of circadian illuminance are sufficient to entrain and maintain circadian locomotor rhythms in Drosophila
Source: Sci Rep. 2016 Nov 24;6:37784. doi: 10.1038/srep37784 (PMC5121609; doi:10.1038/srep37784)
Supplement: Supplementary Information [file srep37784-s1.pdf]

Cycles of circadian illuminance are sufficient to entrain and maintain circadian locomotor rhythms in *Drosophila*

Eunjoo Cho[1,2*], Ji Hye Oh[3*], Euna Lee[1, 4], Young Rag Do[3#], Eun Young Kim[1,2,4#]

[1]Department of Brain Science, [2]Chronic Inflammatory Disease Research Center, Ajou University School of Medicine, 164 Worldcup-ro, Yeongtong-gu, Suwon, Kyunggi-do, 16499, Republic of Korea. [3]Department of Chemistry, Kookmin University, Seoul, 136-702, Republic of Korea. [4]Neuroscience Graduate Program, BK21 Plus Program, Department of Biomedical Sciences, Ajou University School of Medicine, 164 Worldcup-ro, Yeongtong-gu, Suwon, Kyunggi-do, 16499, Republic of Korea.

*These authors contributed equally to this work.

#Correspondence: Eun Young Kim, Department of Brain Science, Ajou University School of Medicine, 164 Worldcup-ro, Yeongtong-gu, Suwon, Kyunggi-do, 443-380, Republic of Korea. E-mail: ekim@ajou.ac.kr; Young Rag Do, [2]Department of Chemistry, Kookmin University, Seoul, 136-702, Republic of Korea. E-mail: yrdo@kookmin.ac.kr

Supplementary information (SI)

SI 1. Vision and Circadian (non-visual) performance [S1-S4]

SI 1-1. Luminous efficacy of radiation (LER) and circadian LER (CER)

Figure S1 shows the photopic luminous efficacy function (V(λ)) and circadian luminous efficacy function (C(λ)).

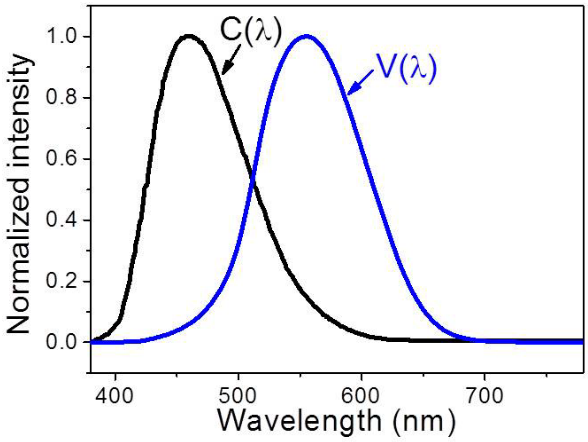

Figure S1. The normalized spectra of the photopic luminous efficacy function (V(λ)) and circadian luminous efficacy function (C(λ)).

The LER is a parameter explaining theoretically how bright the radiation of the emission spectrum is perceived by the average human eye. The LER was calculated with photopic spectral luminous efficacy function (V(λ)) and the SPDs of light source (S(λ)) (See Eq. S1). The maximum value of the LER is 683 lm/W with SPD of 555 nm green monochromatic light.

$$\mathrm{LER\ (lm/W)} = 683(\mathrm{lm/W})\frac{\int \mathrm{V}(\lambda)\mathrm{S}(\lambda)\mathrm{d}\lambda}{\int \mathrm{S}(\lambda)\mathrm{d}\lambda} \qquad \text{Eq. S1}$$

Similarly, the CER is a parameter explaining theoretically how circadianly (non-visually) bright the radiation of emission spectrum is perceived by the average human eye. The CER was calculated with circadian spectral luminous efficacy function (C(λ)) and the SPDs of light source (S(λ)) (See Eq. S2). The maximum value of the CER is 683 blm/W with SPD of 460 nm blue monochromatic light.

$$\mathrm{CER\ (b\,lm/W)} = 683(\mathrm{blm/W})\frac{\int \mathrm{C}(\lambda)\mathrm{S}(\lambda)\mathrm{d}\lambda}{\int \mathrm{S}(\lambda)\mathrm{d}\lambda} \qquad \text{Eq. S2}$$

SI 1-2. Circadian action factor (CAF)

The ratio of CER to LER indicates the CAF (Eq. S3). This value means the circadian response (biological action) per unit of vision response.

$$\mathrm{CAF\ (blm/lm)} = \frac{\mathrm{CER\ (blm/W)}}{\mathrm{LER\ (lm/W)}} \qquad \mathrm{Eq.\ S3}$$

The correlated color temperature and color, especially blue, are mostly effect on the circadian performance. The daylight, CCT of 6,500 K, has CAF value around 1.00. High CCT lamp (bluish white) has high CAF value and low CCT lamp (reddish white) has low CAF value.

SI 1-3. Illuminance (VIL) and circadian illuminance (CIL) [S3, S4]

The VIL is one of the yardsticks for measuring the brightness of light. The VIL means total amount of luminous flux per unit area from the incident light. As shown in Eq. S4, the CIL is calculated from the CAF and VIL. The CIL means total amount of circadian luminous flux per unit area from the incident light.

$$\mathrm{CIL\ (blm/m^2\,, blx)} = \mathrm{CAF\ (blm/lm)}\ \times \mathrm{VIL\ (lm/m^2\,, lx)} \qquad \mathrm{Eq.\ S4}$$

The visual illuminance (VIL) values were changed by the brightness, color, or CCT of light source. Therefore, the VIL of different light sources show different values even though the applied power of light source has the same input power. This means that if we use the VIL value as a yardstick for indicating brightness effect on human health, such as melatonin suppression value, we need to set the values of every light source. In contrast, the CIL value can be changed by only brightness of light source and the CIL shows almost same value regardless of light sources. Therefore, the CIL value is one of good yardsticks for indicating brightness effect on human health.

SI 2. Fabrication of monochromatic green, amber, and red phosphor-converted LEDs. [S3, S5]

To fabricate the monochromatic green(G), amber(A), and red(R) phosphor-converted LEDs (pc-LEDs), InGaN blue LEDs (λ_{max}=445 nm) were used as an excitation source and phosphors (G: $(Ba,Ca,Sr)_nSiO_4$:Eu, A: $(Ba,Ca,Sr)_3SiO_5$:Eu, R: $(Sr,Ca)AlSiN_3$:Eu). Phosphor paste, mixed with silicone binder and phosphors, were injected into B LED cup and hardened in an oven. After then, the long-wavelength pass dichroic filters (LPDFs) were capped on the G, A, and R pc-LEDs to realize monochromatic color by reflecting blue excitation source not to color mixing with excitation source and phosphor emission source. The fabrication methods for LPDFs were explained in our previous work.[S5] Figure S2 shows the emission SPD and 1931 CIE color coordinates for B LED, and LPDF-capped monochromatic G, A, and R pc-LEDs

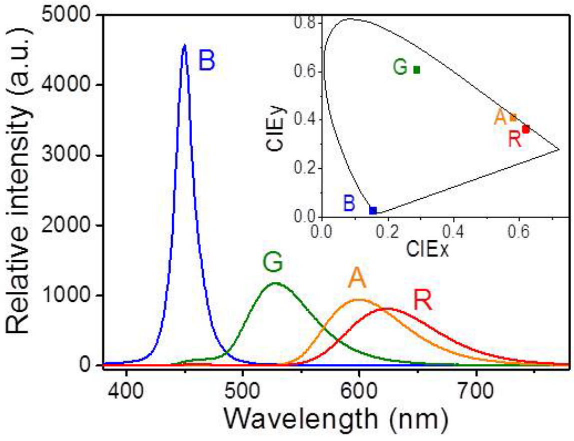

Figure S2. The relative emission SPD and 1931 CIE color coordinates (inset) of B semiconductor LED and G, A, and R pc-LEDs.

References

[S1] Žukauskas A, Vaicekauskas R, Vitta P. Optimization of solid-state lamps for photobiologically friendly mesopic lighting. *Appl Opt.* 2012; **51**:8423-8432.

[S2] Gall D, Bieske K. Definition and measurement of circadian radiometric quantities. *In Proceedings of the CIE symposium 2004 on Light and Health: Non-visual effects* 2004; **S**:129-132.

[S3] Oh JH, Yang SJ, Do YR. Healthy, natural, efficient and tunable lighting: four-package white LEDs for optimizing the circadian effect, color quality and vision performance. *Light: Sci. Appl.* 2014; **3**:e141.

[S4] Oh JH, Yoo H, Park HK, Do YR. Analysis of circadian properties and healthy levels of blue light from smartphones at night. *Sci. Rep.* 2015; **5**:11325.

[S5] Oh JR, Cho S-H, Park HK, Oh JH, Lee Y-H, Kim W, Do YR. The realization of a whole palette of colors in a green gap by monochromatic phosphor-converted light-emitting diodes. *Opt. Express* 2011;**19**:4188-4198.

SI 3. Supplementary figure legend

Figure S1. Molecular oscillations of PER in w^{1118} flies were similar to those in CS flies despite different patterns of circadian locomotor activity.

Head extracts of CS and w^{1118} flies were collected at indicated time points on day 5 of the CH/CL cycle and analyzed by immunoblotting with anti-PER antibody. Actin was used as a loading control. ZT, zeitgeber time.

Figure S2. Molecular oscillations of PER are dampened after exposure to constant CL light.

CS flies were first exposed to LL, entrained to CH/CL cycles for 7 days, and then exposed to constant CL light. Heads of CS flies were collected on day 5 of CH/CL and day 2 of constant CL at indicated time points. Head extracts were obtained and analyzed by immunoblotting with anti-PER antibody. Actin was used as a loading control. ZT, zeitgeber time.

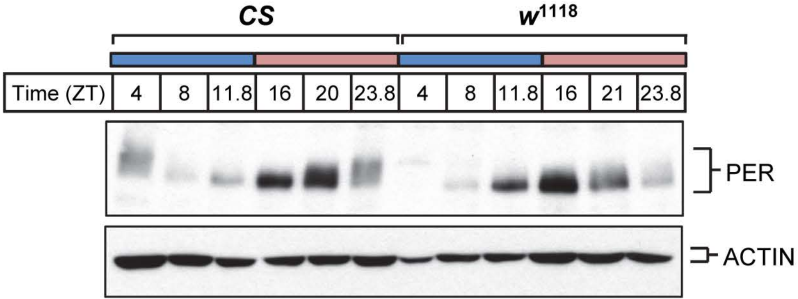

CS
w^{1118}
Time (ZT)
4
8
11.8
16
20
23.8
4
8
11.8
16
21
23.8
PER
ACTIN

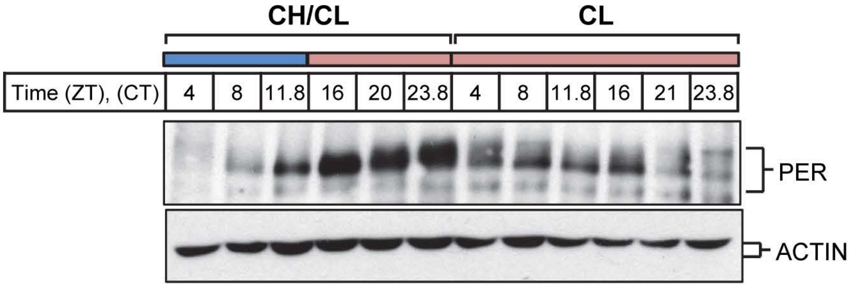

CH/CL
CL
Time (ZT), (CT)
4
8
11.8
16
20
23.8
4
8
11.8
16
21
23.8
PER
ACTIN
